# Microglial control of neuronal activity

**DOI:** 10.3389/fncel.2013.00032

**Published:** 2013-03-28

**Authors:** Catherine Béchade, Yasmine Cantaut-Belarif, Alain Bessis

**Affiliations:** Institut de Biologie, Ecole Normale Supérieure, Inserm U1025, CNRS UMR8197Paris, France

**Keywords:** microglia, neurotransmission, inflammation, synapse, glial cells

## Abstract

Fine-tuning of neuronal activity was thought to be a neuron-autonomous mechanism until the discovery that astrocytes are active players of synaptic transmission. The involvement of astrocytes has changed our understanding of the roles of non-neuronal cells and shed new light on the regulation of neuronal activity. Microglial cells are the macrophages of the brain and they have been mostly investigated as immune cells. However, recent data discussed in this review support the notion that, similarly to astrocytes, microglia are involved in the regulation of neuronal activity. For instance, in most, if not all, brain pathologies a strong temporal correlation has long been known to exist between the pathological activation of microglia and dysfunction of neuronal activity. Recent studies have convincingly shown that alteration of microglial function is responsible for pathological neuronal activity. This causal relationship has also been demonstrated in mice bearing loss-of-function mutations in genes specifically expressed by microglia. In addition to these long-term regulations of neuronal activity, recent data show that microglia can also rapidly regulate neuronal activity, thereby acting as partners of neurotransmission.

## Introduction

Microglial cells are one of the glial cell populations of the brain. In contrast to other glial cell types such as oligodendrocytes or astrocytes, the role of microglia in the regulation of neuronal activity has been somewhat overlooked. Microglia are macrophages of the nervous tissue and as immune cells they can detect and react to infection, trauma, ischemia, degeneration, or any alterations in brain homeostasis. Actually, most brain pathologies, if not all, are associated with early microglial activation^1^. Thus, microglial activation was demonstrated based on histopathological data, *in vivo* brain imaging or cytokine expression upon axotomy (Blinzinger and Kreutzberg, 1968), during degenerative (Haga et al., 1989; Cagnin et al., 2001; reviewed in Cameron and Landreth, 2010) or neuropsychiatric diseases (review in Beumer et al., 2012). Of note, the above-described disorders are also associated with early synaptic dysfunction (Blinzinger and Kreutzberg, 1968; references in Selkoe, 2002; Penzes et al., 2011; Peça and Feng, 2012). Such a temporal correlation between microglial activation and synaptic dysfunction during brain pathologies suggests that regulatory interactions exist between the activation of microglia and neurotransmission. In addition, the functional properties of microglia are compatible with an involvement in the control of neuronal activity. They express receptors for most neurotransmitters (Kettenmann et al., 2011; Kaindl et al., 2012) and produce a large repertoire of molecules known to modulate neuronal activity and plasticity. In addition, microglia are highly ramified cells and their ramifications rapidly scan the local environment and react to its modification (Davalos et al., 2005). Finally, microglial processes physically contact synaptic elements (Wake et al., 2009; Tremblay et al., 2010; see also Schafer et al., 2012), allowing for an accurate control of synaptic function.

In this review, we will highlight recent studies suggesting or demonstrating the involvement of microglia in the control of neuronal activity. Firstly, we will describe how microglial dysfunction is primarily responsible for the alterations in neuronal activity under pathological situations. We will then show that in the healthy brain microglia can be described as partners of neurotransmission.

## Microglia dysfunction perturbs neuronal activity

Microglia were initially described as sensors of pathological events (Kreutzberg, 1996). It is now widely accepted that microglia are not only sensors but also active players of pathological states in the brain. Understanding the consequences of microglial dysfunction on neuronal phenotype is important to understand the etiology of the disease state and to propose therapeutic strategies. In this first section we will review studies in which microglia are the primary cause of alterations in neuronal activity during non-physiological states. Importantly, the information gathered from pathological situations is relevant for the understanding microglial function in the absence of pathology, as will be discussed in the second section of this review.

Analyses of mice bearing loss-of-function mutations in genes involved in microglia-specific pathways exemplify the link between microglial dysfunction and neuronal activity. CX3CR1 is the microglial receptor for the neuronal chemokine fractalkine (CX3CL1). This complementary expression of ligand and receptor on neurons and microglia respectively, suggests that their interaction may play a role in modulating neurotransmission. Mice with a CX3CR1 loss-of-function mutation exhibit an impairment of hippocampal long-term potentiation (LTP) as well as cognitive deficits (Rogers et al., 2011). The CX3CL1/R1 signaling pathway also appears to be involved in synaptic maturation since CX3CR1 deficiency leads to a delay in the maturation of glutamatergic thalamocortical synapses, as well as a transient immature connectivity in the developing hippocampus (Paolicelli et al., 2011; Hoshiko et al., 2012). Of note, these latter alterations might be secondary to a decreased recruitment of microglia and not to a direct involvement of CX3CR1 signaling in the regulation of neurotransmission (Paolicelli et al., 2011; Hoshiko et al., 2012). Another example of a neuronal-microglial interaction is provided by the analysis of CD200-deficient mice. CD200R is a membrane protein exclusively expressed by microglia. Its ligand, CD200 is expressed by neurons, oligodendrocytes and astrocytes (Costello et al., 2011). It was demonstrated that LTP is inhibited in CD200-deficient mice, further supporting the notion that the integrity of microglial signaling is crucial for neurotransmission homeostasis (Costello et al., 2011). Finally, synaptic alterations have also been demonstrated upon the loss-of-function mutation of DAP12, a transmembrane protein associated with various lymphoid and myeloid receptors such as TREM2 (Tomasello et al., 2000). In the brain, DAP12 and TREM2 are exclusively expressed by microglia and DAP12 loss-of-function results in an enhanced hippocampal LTP and major changes in glutamatergic transmission (Roumier et al., 2004, 2008). As for CX3CR1- and CD200-deficient mice, the molecular mechanisms linking microglial deficiency to synaptic alterations in DAP12KO mice are not known. Interestingly however, the DAP12-mutant mouse is a model for Nasu-Hakola disease in which patients display progressive presenile dementia associated with bone cysts (Hakola, 1972), together with leukodystrophy and astrogliosis in the brain (Satoh et al., 2011). Nasu-Hakola disease is caused by mutations in the genes encoding microglial DAP12 or TREM2 (Paloneva et al., 2000), and because of this restricted expression, it has been described as the first microgliopathy (Bianchin et al., 2010). Thus, dysfunction of DAP12 signaling, which is exclusively expressed by microglia impacts synaptic transmission (Roumier et al., 2004), mouse behavior (Kaifu et al., 2003), and higher brain functions in human (Paloneva et al., 2000).

A link between microglia and higher brain function has also been proposed in the case of the mouse model of obsessive-compulsive disorder. Disruption of the Hoxb8 gene, expressed by a subpopulation of microglia, caused mice to groom compulsively (Chen et al., 2010; see however Holstege et al., 2008). Transplantation of wild type bone-marrow cells into Hoxb8 mutant mice rescued the phenotype (Chen et al., 2010) leading to the hypothesis that the pathological grooming behavior observed in Hoxb8 mutant mice may result from deficient mutant microglia.

Rett syndrome is another example of microglial involvement in psychiatric disease. Rett syndrome is an autism spectrum disorder caused by mutations in the gene encoding the methyl CpG binding protein-2 (MeCP2). Rett syndrome patients exhibit dendritic and synaptic abnormalities in selected regions (references in Chahrour and Zoghbi, 2007). MeCP2 deficient mice mimic the human syndrome (Chen et al., 2001; Guy et al., 2001; Shahbazian et al., 2002). Transplantation of wild type bone marrow into irradiated MeCP2-null hosts was recently shown to lead to engraftment of MeCP2-expressing microglia in the brain parenchyma and to a rescue of the brain phenotype (Derecki et al., 2012). Involvement of microglia in Rett syndrome is strengthened by *in vitro* observations showing that MeCP2-null microglia release high levels of glutamate, which induced changes in dendritic morphology and a reduced number of postsynaptic densities (Maezawa and Jin, 2010). Thus, microglia have an active role in this disorder by a mechanism that remains to be described.

These examples of psychiatric phenotypes induced primarily by deficiencies of microglial function support the notion that microglia can actively modulate neuronal functions, including learning and memory (Blank and Prinz, 2013). Yet, it cannot be excluded that microglial dysfunctions induce a general change of brain homeostasis resulting in non-specific defects in neuronal activity. However, in some instances, such as chronic pain, it could be shown that pathological effects on neuronal activity are due a deregulation of local microglial mechanisms that might be dedicated to the control of neurotransmission. For instance, stimulation of microglial P2X4 receptors induces the release of pain mediators such as PGE2 (Ulmann et al., 2010) or BDNF (Coull et al., 2005) and is necessary for the induction of allodynia after nerve injury (Tsuda et al., 2003). In addition, upon neuropathic pain, the dorsal horn microglia produces BDNF, which stimulates the neuronal TrkB receptor and induces a shift in the chloride gradient in nociceptive neurons (Coull et al., 2005). Such shift increases the excitability of the neurons through GABA_A_ receptor-mediated depolarization (Coull et al., 2003).

These examples show that local and specific interactions between microglia and neurons can be responsible for the altered neuronal activity observed in pathology. However, microglia and neurons functionally interact in healthy conditions (Tremblay et al., 2011) as well, and several studies have now established that microglia can rapidly modulate neuronal activity in basal conditions.

## Microglia as genuine partners of synaptic activity

Under physiological conditions, microglia react rapidly to neuronal activity by modulating the physical contacts that their numerous processes continuously establish with synaptic elements (Wake et al., 2009; Tremblay et al., 2010). Microglia are thus potentially accurate sensors of neuronal activity and a reciprocal control of neurotransmission by microglia can be expected. The ability of microglia to rapidly modulate synaptic activity was initially exemplified by treating cultured neurons or acute brain slices with medium conditioned by cultured microglia. Microglia conditioned-medium was shown to increase both the amplitude and duration of the NMDA-receptor induced currents (Moriguchi et al., 2003; Hayashi et al., 2006). The nature of the signaling molecules involved in this process is still unknown and were proposed to be a secreted protein(s) (Moriguchi et al., 2003) or glycine (Hayashi et al., 2006). In fact, microglia produce a broad spectrum of signaling molecules known to regulate synaptic function, including cytokines (Elkabes et al., 1996; Hanisch, 2002), neurotransmitters (Piani and Fontana, 1994; Hayashi et al., 2006; Flierl et al., 2007; Pascual et al., 2012), and extracellular matrix proteins (Chamak et al., 1994). A direct regulation of synaptic properties by microglia is therefore expected. Amongst the microglial molecules with a putative role in neurotransmission, TNFα deserves specific attention. This cytokine was shown to control basal synaptic functions (Santello et al., 2011) as well as plasticity (Stellwagen and Malenka, 2006; Kaneko et al., 2008; Costello et al., 2011; Santello et al., 2011), and this role was attributed to TNFα produced by astrocytes (Stellwagen and Malenka, 2006). However, the astrocytes have often been thought to express TNFα because cultures of astrocytes are consistently contaminated by microglia (Saura, 2007; Barres, 2008). In addition, the transcriptome analysis from purified astrocytes reveals no TNFα-encoding transcript in astrocytes (Sharma et al., 2007; Cahoy et al., 2008; Doyle et al., 2008; Meissner et al., 2008; Foo et al., 2011; Zamanian et al., 2012). Thus, the TNFα that controls several aspects of synaptic transmission might, in fact, be produced by microglial cells, but this has not yet been firmly established.

It has also been shown that microglia can shed micro-vesicles a few seconds after ATP stimulation, most probably by a P2X7-dependent mechanism (Bianco et al., 2005). When these vesicles were harvested from cultured microglia and applied to cultured hippocampal neurons, they induced an increased frequency of miniature excitatory post-synaptic currents (mEPSC), supposedly through presynaptic regulation (Antonucci et al., 2012). Analysis of the regulatory pathway between microglia and synaptic activity led the authors to propose that microglial micro-vesicles regulate mEPSCs through a phosphatydil-dependent regulation of presynaptic vesicle release (Antonucci et al., 2012). The functional relevance and specificity of this mechanism remains to be established but it raises the provocative hypothesis that physical contacts, or membrane exchange between microglia and neurons, could actively and rapidly regulate neurotransmission.

**Figure 1:**
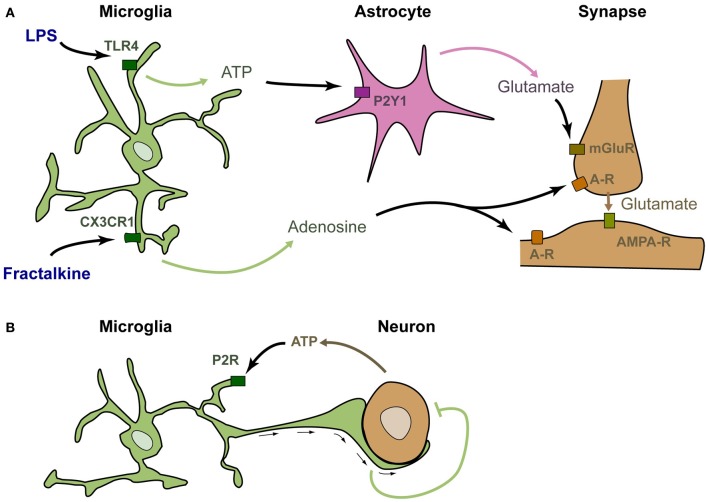
**Microglia are genuine partners of synaptic transmission. (A)** In acute rodent brain slices, stimulation of microglia by LPS induces the rapid release of ATP, which recruits astrocytes. Upon purinergic stimulation, astrocytes release glutamate, inducing a mGluR-dependent release of presynaptic glutamate (Pascual et al., 2012). Stimulation of microglia by fractalkine induces the release of adenosine, which decreases neuronal activity (Meucci et al., 1998; Ragozzino et al., 2006; Piccinin et al., 2010). **(B)** In zebrafish larva, active neurons release the ATP that attracts microglial bulbous processes. These processes decrease neuronal activity by an as yet unknown mechanism (Li et al., 2012).

The above-described studies suggest, but do not demonstrate, that microglia can rapidly modulate synaptic function. Several studies have specifically stimulated microglia and analyzed the consequences on neuronal activity in a similar way to what was done to investigate the role of astrocytes in neurotransmission. Application of fractalkine onto neuron cultures was shown to induce a strong and rapid modulation of calcium currents in neurons (Meucci et al., 1998). Such modulation was actually the first demonstration that stimulation of microglia could rapidly modulate the activity of neurons (although it was at first incorrectly attributed to a direct stimulation of neurons by fractalkine). This modulation has also been confirmed in acute hippocampal slices, in which stimulation of microglia by fractalkine induces a significant and transient reduction of the amplitude of evoked EPSCs in CA1 pyramidal neurons (Ragozzino et al., 2006; see Figure 1A). It was further demonstrated that this reduction involves adenosine, supposedly acting on neuronal A3R receptors (Piccinin et al., 2010). The probable mechanism of regulation is that fractalkine induces the microglial release of adenosine, which in turn inhibits the presynaptic release of glutamate (Figure 1A). Alternatively, microglia could produce ATP that is rapidly degraded into adenosine by ectonucleotidases. The involvement of other cell types such as astrocytes has not yet been ruled out.

An alternative rapid regulation of neuronal activity by microglia has recently been established upon application of lipo-polysaccharide (LPS—Pascual et al., 2012; Figure 1A). LPS is a ligand of TLR4 that mimics bacterial infection and can reveal pathological pathways. TLR4 is exquisitely expressed by microglia and can also be stimulated by several endogenous ligands (Habich et al., 2005; Gondokaryono et al., 2007; Midwood et al., 2009; Milanski et al., 2009; references in Lucin and Wyss-Coray, 2009). Therefore, the mechanisms revealed by LPS application probably have a physiological relevance. Stimulation of microglia by addition of LPS onto acute hippocampal slices induces a rapid and transient increase in the frequency of spontaneous synaptic AMPAergic post-synaptic currents in CA1 neurons. This effect does not occur in slices prepared from Pu.1 deficient mice that lack microglia, showing that the effect of LPS requires microglia. It was then demonstrated that upon LPS stimulation, microglia rapidly produce ATP, which recruits astrocytes. Astrocytes subsequently release glutamate, and this leads to increased excitatory transmission via a metabotropic glutamate receptor-dependent mechanism (Pascual et al., 2012).

The above-described studies show that stimulation of microglia modulates neuronal activity *in vitro*. The occurrence of regulating interactions between microglia and neuronal activity has recently been demonstrated *in vivo* in the zebrafish larva (Li et al., 2012; Figure 1B). In this system, microglia monitor spontaneous or visually evoked neuronal activity, and send bulbous processes toward the most active neurons, as detected by their production of ATP. These contacts between microglial endings and active neurons induce a rapid decrease in both frequency and amplitude of neuronal calcium events (Li et al., 2012). This study confirms and extends the data obtained upon stimulation of microglia and further demonstrates that microglia are genuine partners of neuronal activity in the healthy brain.

## The right tools to target the right cells

The role of microglia in the regulation of neurotransmission is far less studied than that of astrocytes. This might be due to a lesser involvement of microglia in such regulation. Alternatively, this could also be due to the fact that the characterization of microglia as regulators of neurotransmission has been hindered by the lack of tools to specifically stimulate or block their function. Such tools are available for astrocytes and their function has been blocked by application of pharmacological inhibitors such as Fluoroacetate or calcium chelators (Henneberger et al., 2010). Stimulation of astrocytes has also been achieved, mechanically (see e.g., Liu et al., 2011) or by local application of synthetic agonists or local uncaging of calcium or glutamate (Pascual et al., 2005; Agulhon et al., 2010). Although the physiological relevance of such treatments is still debated (Hamilton and Attwell, 2010) these protocols allowed the characterization of astrocytes as regulators of the normal function and plasticity of neural circuits *in vitro* and *in vivo*. Comparable tools to tune the function of microglia are lacking, mostly because of a specificity issue. For instance, minocycline is known to block microglial function (Yrjänheikki et al., 1998), but its molecular and cellular targets remain unidentified and its specificity remains to be firmly established. Moreover, microglia can be stimulated by a large variety of inflammatory molecules such as cytokines or interleukins, but their receptors have also been detected on neurons and astrocytes, preventing accurate interpretation of their putative effects. In addition, as mentioned previously in relation to the cellular origin of TNFα, the consistent contamination of neuronal and astrocyte cultures by microglia has made it difficult to address the correct expression of microglial molecules. For instance, CX3CR1, the fractalkine receptor that was initially thought to be expressed by neurons (Meucci et al., 1998, 2000; Hughes et al., 2002; Ragozzino et al., 2006), is now demonstrated to be exclusively expressed by microglia (Cardona et al., 2006; Lauro et al., 2008). Similarly, TLR4, the LPS receptor, was mistakenly detected in astrocytic (Bowman et al., 2003; Alfonso-Loeches et al., 2010) and neuronal cultures (Tang et al., 2007). Indeed, when microglia were efficiently depleted from astrocyte cultures, TLR4 was no longer detected (Lehnardt et al., 2002; Pascual et al., 2012). In addition, expression of TLR4 has never been found in healthy neurons or astrocytes. Finally, data mining of Gene Expression Omnibus DNA array experiments performed on purified cells confirmed that TLR4 is exclusively expressed by microglia (Pascual et al., 2012). Thus, CX3CR1 and TLR4 expression is limited to microglia and, as described above, can be used to specifically stimulate these cells and study their involvement in biological processes. We speculate that the future development tools to specifically block microglial function will also be instrumental to understand the involvement of these cells in wide variety of physiological processes.

## Conclusion

The biological relevance of microglia as active sensors of brain parenchyma was until recently, principally recognized in pathological tissues. The role of microglia in the healthy brain is now acknowledged (Graeber, 2010; Pont-Lezica et al., 2011; Tremblay et al., 2011). Here we have reviewed studies indicating that microglia are able to control neuronal activity, from synaptic transmission to higher brain functions. Microglia have often been described as “good” or “bad” cells (Kempermann and Neumann, 2003; Kettenmann, 2007; Watkins et al., 2007; Aguzzi et al., 2013). Considering microglia as partners of neuronal function will certainly help to provide a more accurate and integrated understanding of their roles, beyond the primary “beneficial vs. detrimental” dichotomy. It will also extend our understanding of non-cell autonomous regulation of neuronal activity and shed new light on the role of microglia in the pathological brain.

### Conflict of interest statement

The authors declare that the research was conducted in the absence of any commercial or financial relationships that could be construed as a potential conflict of interest.

## Footnotes

^1^The notion of “activation” of microglia is quite a loosely-defined concept adapted from the well-defined concept of macrophage activation (Taylor et al., 2005; Perry et al., 2007). Activation is associated with pathology but is often used to describe the consequences of any stimulation of microglia. Because different stimulation can induce different responses in microglia, there is not one single parameter that characterizes an “activation.” Therefore, various parameters have been used to establish microglial “activation” such as changes in density, morphology, or expression of proteins. Whenever possible, we have used the notion of stimulation instead of activation and described the nature of the stimuli.
